# Comparing the efficacy of a web-assisted calprotectin-based treatment algorithm (IBD-live) with usual practices in teenagers with inflammatory bowel disease: study protocol for a randomized controlled trial

**DOI:** 10.1186/s13063-015-0787-x

**Published:** 2015-06-16

**Authors:** Anke Heida, Alie Dijkstra, Henk Groen, Anneke Muller Kobold, Henkjan Verkade, Patrick van Rheenen

**Affiliations:** Department of Pediatric Gastroenterology, University of Groningen, University Medical Centre Groningen, PO Box 30001, 9700 RB Groningen, The Netherlands; Department of Clinical Epidemiology (Medical Technology Assessment), University of Groningen, University Medical Centre Groningen, Hanzeplein 1, 9713 GZ Groningen, The Netherlands; Department of Laboratory Medicine, University of Groningen, University Medical Centre Groningen, Hanzeplein 1, 9713 GZ Groningen, The Netherlands

**Keywords:** Children, Inflammatory bowel disease, Crohn’s disease, Ulcerative colitis, Self-monitoring, Calprotectin, Telemedicine, E-health

## Abstract

**Background:**

To prevent clinical relapse in teenagers with inflammatory bowel disease (IBD) there is a need to monitor disease activity continuously. Timely optimisation of medical treatment may nip a preclinical relapse in the bud and change the natural course of IBD. Traditionally, disease monitoring is done during scheduled visits, but this is when most teenagers report full control. IBD care could be more efficient if patients were seen at times of clinical need. This study aims to examine the effectiveness of a web-assisted calprotectin-based treatment algorithm (IBD-live) compared with usual practices in teenagers with IBD.

**Methods/design:**

A randomized trial of web-based disease monitoring versus usual care is conducted at 10 Dutch IBD care centers. We plan to recruit 180 patients between 10- and 19-years old with quiescent IBD at baseline. Teenagers assigned to IBD-live will use the flarometer -an automatic cumulation of disease activity and fecal calprotectin measurements- to estimate probability of relapse. In case the flarometer indicates high risk the patient requires treatment intensification in accordance with national guidelines; low risk means that maintenance therapy is unchanged; and intermediate risk requires optimisation of drug adherence. Patients assigned to usual practice get the best accepted medical care with regular health checks. Primary outcome is the frequency of relapse at 52 weeks of follow-up. The diagnosis of relapse is based on a clinical activity index score >10 points necessitating remission induction therapy. Secondary outcomes include quality of life and cost-effectiveness.

**Discussion:**

Web-assisted monitoring of disease activity with rapid access for those with acute relapse may allow teenagers to develop skills that are required of adult patients (including communication and self-determination). Similar monitoring systems have been introduced for teenagers with asthma and diabetes, with a positive effect on disease control, but the intervention has not been evaluated in teenagers with IBD. A randomized trial in adult patients with ulcerative colitis showed that a web-assisted treatment algorithm is feasible, safe and cost-effective. Results of the current trial are expected to have important implications for teenagers with IBD that incurs substantial health burdens and economic costs.

**Trial registration:**

Dutch Trial Register identifier: NTR3759 (registered 29 December 2012)

## Background

Inflammatory bowel diseae (IBD) includes two major forms of intestinal inflammation: Crohn’s disease and ulcerative colitis. In both types inflammation waxes and wanes over time in an unpredictable fashion. Treatment of children and adolescents with IBD is aimed at inducing and maintaining remission of disease activity to ensure normal growth and pubertal development, and improving the quality of life of patients. To prevent relapses there is need for continuous monitoring of disease activity for timely optimisation of medical treatment.

Twenty five per cent of the total IBD cases present in childhood or adolescence [1], and the incidence is increasing [2–4]. In the Netherlands each year approximately 250 teenagers are diagnosed with IBD [5]. Health professionals confronted with the increased disease burden may be interested in finding ways to ease the pressure on overstretched clinics with new approaches to monitor disease activity in IBD.

### Existing knowledge

Endoscopy is the standard for assessing intestinal inflammation [6], but the invasive nature of the procedure limits its use for routine evaluation of disease activity. Intensification of treatment in teenagers is therefore guided by regular use of clinical composite scores including the Pediatric Crohn’s Disease Activity Index (PCDAI) [7] and the Pediatric Ulcerative Colitis Activity Index (PUCAI) [8]. When used as stand-alone tests these scores may be subject to bias. High scores may reflect relapse, but could also be caused by gastrointestinal symptoms not related to IBD, such as irritable bowel syndrome. Moreover, underreporting the symptom burden is a characteristic coping strategy for teenagers during a face-to-face conversation with a physician [9]. Traditionally, many clinicians use C-reactive protein (CRP) as an objective add-on test to monitor disease activity [10].

Fecal calprotectin (fCal) is a noninvasive marker of inflammation that correlates well with endoscopic and histopathological disease activity [11]. Measuring fCal is a useful screening tool for identifying patients who are most likely to need endoscopy for suspected IBD [12]. We assessed the value of periodic CRP and fCal testing to predict early relapse in teenagers with confirmed IBD [13]. We followed 62 patients who claimed to have no symptoms and found that fCal levels increase prior to CRP and before manifestations of clinical symptoms. The positive predictive value to detect early relapse was 40 % with CRP and 60 % with fCal testing. We concluded that fCal is a better add-on test to monitor disease activity than CRP. Periodic use of fCal could help to identify teenagers who require treatment intensification at the time of minimal disease rather than at the time of clinically overt relapse.

### Home monitoring of disease activity and treatment algorithm

**Fig. 1:**
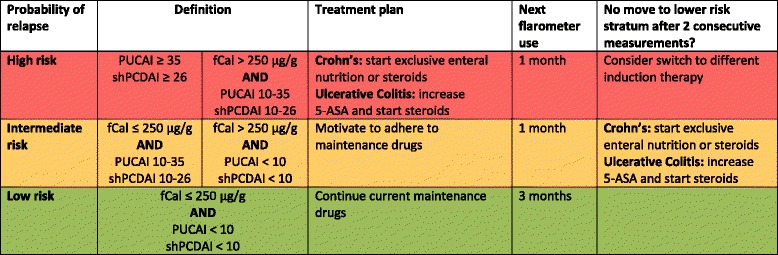
Flarometer home monitoring strategy and treatment algorithm

**Fig. 2:**
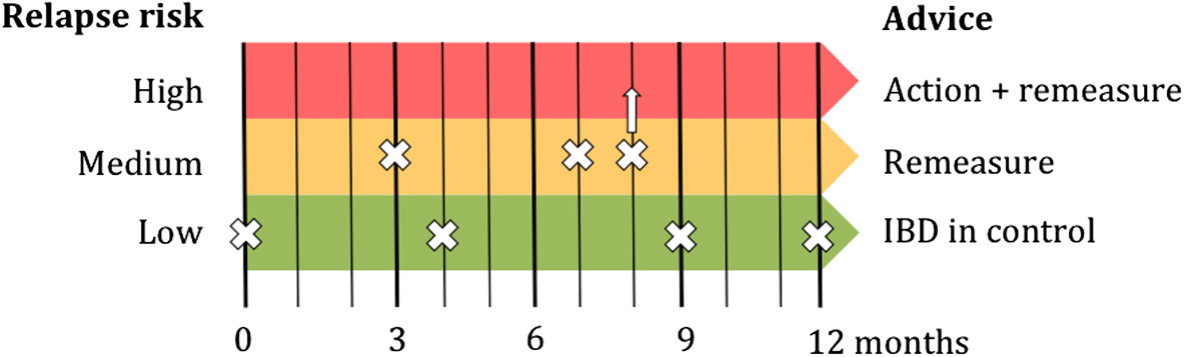
Graphical representation of flarometer test frequency and treatment advice. In the high-risk stratum, clinical relapse is suspected and the local IBD-team is advised to intensify treatment and remeasure in one month. In the low-risk stratum, maintenance therapy remains unchanged and an alert to use the flarometer will be sent in three months. In the intermediate range, drug adherence is optimised and the flarometer will be used again in a month. After two consecutive intermediate results the local IBD-team is advised to intensify treatment. This scenario is shown at eight months. *IBD* Inflammatory bowel disease

The above described “Full Control Study” [13] provided evidence that disease activity scores and fCal measurements are the best combination to predict relapse in teenagers who are doing well. Building on this we designed a relapse risk stratification tool (flarometer, Figure 1) that can be used at home. The flarometer is an automatic cumulation of patients’ answers to disease activity questionnaires (PUCAI or the short version of the PCDAI (shPCDAI) [14], which excludes blood test results). After completion of these questionnaires the web application asks the patient to send a stool sample to the Department of Laboratory Medicine of the University Medical Centre Groningen, where it is analysed for fCal with a rapid test. In case the PUCAI or shPCDAI indicates moderate to severe disease activity, the patient is automatically advised not to await the stool result, but to immediately contact the local IBD-team. The stool result is entered into the flarometer system within 24 hours after reception, and a customized clinical alert is transmitted to the patient and local IBD-team. The advice will vary with the position of the pointer on the flarometer scale. When the pointer is in the high-risk stratum, clinical relapse is highly suspected and the local IBD-team is advised to intensify treatment in accordance with national guidelines [15]. In the low-risk stratum maintenance therapy remains unchanged and an alert to use the flarometer will be sent in three months. In the intermediate range, drug adherence is optimised and the flarometer will be used again in a month. After two consecutive intermediate results the local IBD-team is advised to intensify treatment (Fig. 2).

### Need for a trial

Web-assisted monitoring of disease activity with rapid access for those with acute relapse may allow teenagers to develop skills that are required of adult patients (including communication and self-determination) [16]. A randomized trial in adult patients with ulcerative colitis showed that a web-assisted treatment algorithm is feasible, safe and cost-effective compared to usual care. The intervention increased compliance and quality of life, and reduced healthcare costs [17]. Web-based disease monitoring has been introduced for teenagers with asthma [18] and diabetes [19], with a positive effect on disease control, but the intervention has not been evaluated in teenagers with IBD.

The trial described in this protocol aims to examine the efficacy of a web-assisted calprotectin-based treatment algorithm, compared with usual practice, on disease course among teenagers with IBD. This project will move IBD care for teenagers into a new era, from traditional paternalism (the doctor at the wheel) to a phase in which teenagers take ownership of their chronic disease and participate in the therapeutic decision-making process (teenagers at the wheel). Informing teenagers periodically about fCal results may enable them to understand better the importance of adherence. Studies in adults report that 30–45 % of patients do not use maintenance drugs regularly [20], and nonadherence among teenagers is even higher [21]. Nonadherent patients have an increased risk of clinical relapse [22] and generate greater annual health care costs than adherent patients [23]. Furthermore, the IBD-team could be used more efficiently if patients were seen at times of clinical need, instead of during three-monthly routine visits.

### Choice of comparator

Treatment in the group assigned to usual practices represents the currently best-accepted IBD care. For teenagers with IBD in remission this means regular checks at the outpatient clinic as before the trial. A health check includes a physicians’ rating of disease activity and blood sampling. In case of clinical relapse the patient is advised to intensify treatment in accordance with national guidelines [15]. When a patient experiences a relapse between two regular health checks, rapid access to specialist care is provided.

### Study objectives

We aim to compare the effect of treatment delivered according to a web-assisted calprotectin-based algorithm (IBD-live) against the same treatment delivered according to usual practice.

We hypothesize that use of IBD-live may reduce the relapse rate during a 52-week follow-up period. Furthermore, we postulate that users of IBD-live have higher scores on the disease specific quality of life IMPACT-III questionnaire, and that use of IBD-live is accompanied by lower costs of disease management as compared to usual care.

### Trial design

This trial is designed as a randomized, controlled, open label multicenter superiority strategy trial with two parallel groups. For allocation concealment, randomization will be carried out using a separate list of computer-generated random allocation numbers for each participating center (involving permuted blocks with a ratio of 1:1 allocation).

## Methods—participants, interventions, and outcomes

### Study setting

Study participants will be recruited from six tertiary care centers in the Netherlands (Groningen, Amsterdam (2x), Rotterdam, Utrecht and Leiden), and from four large general teaching hospitals (The Hague, Zwolle, Enschede and Arnhem). The principal investigators at the various sites are members of the Kids with Crohn’s and Colitis (KiCC) working group for Collaborative Research in the Netherlands. Together they treat about two-thirds of the total pediatric IBD population in the Netherlands.

Since 2007 national treatment guidelines for IBD are in use that have provided uniformity in treatment [15]. Immunomodulation with thiopurines is the first choice in patients with Crohn’s disease. In case of failure or intolerance methotrexate can be used. In ulcerative colitis the first choice is aminosalicylate monotherapy. Thiopurine co-medication is recommended in those with frequently relapsing disease. Teenagers with active Crohn’s are treated with steroids and gradual dose tapering, or with an exclusive oral polymeric diet for six weeks. Patients with active ulcerative colitis are treated with steroids and aminosalicylate dose escalation. Anti-tumor necrosis factor (TNF) antibodies are labeled for use after failure of conventional therapy (step-up).

### Inclusion and exclusion criteria for participants

Approximately 10 % of teenagers with IBD will have a severe disease course and require use of the complete arsenal of available therapies in a limited period of time to maintain remission. We aim to include the other 90 % of teenagers with a more stable disease course.

Eligible patients are those: 10- to 19-years old, with quiescent IBD for more than three months before study enrollment, with IBD diagnosed according to the Porto criteria [24] more than six months before enrollment, who have access to internet, with knowledge of the Dutch language, and with an adult caregiver who is willing to actively support participation.

Potential participants will be excluded from the study if any of the following conditions occur: maintenance treatment with infliximab or adalimumab (for unavoidable frequent contact with health providers); presence of ileostomy or ileoanal pouch (as fCal cut-off is not validated for small bowel feces); or any comorbidity at the time of enrollment that requires hospitalization or frequent blood sampling.

### Interventions

Teenagers assigned to the experimental arm of the trial have access to all modules of the web-based portal: (1) the flarometer, an automatic cumulation of an online patient rated PUCAI or shPCDAI, and the result of a fCal point of care test (Quantum Blue® Calprotectin, Bühlmann Laboratories AG, Schönenbuch, Switzerland); (2) a module for direct communication with the local IBD team with guaranteed feedback within two working days; and (3) a module with study questionnaires (quality of life, drug adherence, absenteeism and health care utilization). They will have six monthly health checks at the outpatient clinic to monitor potential adverse effects of the medication. In between these visits teenagers receive alerts to use the flarometer.

The frequency of use depends on the previous flarometer score (Figure 1). When the score is in the high (red) or moderate (orange) risk stratum, next flarometer use is advised after one month. In the low (green) risk stratum an alert to use the flarometer will be sent in three months. Teenagers have the freedom to use the flarometer more often, but fecal samples can only be sent once a month. In case the flarometer is not used despite three reminders (text or e-mail message), the IBD-nurse will contact the teenager by phone.

Both teenager and local IBD team will receive a computer-generated e-mail with the flarometer result, which also includes treatment advice. In the high (red) risk stratum the teenager is advised to contact the local IBD-team for treatment intensification. In case the teenager fails to contact the IBD-team, the IBD-nurse will instead call the teenager. In the low (green) risk stratum maintenance therapy remains unchanged. The intermediate (orange) risk stratum requires evaluation of an imminent relapse, drug adherence and potential intercurrent gastrointestinal infections.

The local IBD-team has the freedom to ignore the flarometer treatment advice, but this should be well-founded and indicated in the patient file. The web-assisted algorithm is certainly not meant to replace the pediatrician. It is intended to guide both pediatricians and patients in joint decisions whether or not to intensify treatment by providing more objective estimates of probability, as a supplement to other relevant clinical information. Any therapeutic intervention remains the responsibility of the treating pediatric gastroenterologist.

### Interventions—modifications

Study participants from both groups are instructed to contact their doctor immediately to report clinical deterioration between regular health checks, or if at any time they want a consultation (side effects, other health related questions). Rapid access to specialist care is guaranteed for both groups. All unscheduled health visits (including those at the IBD clinic, general physician and emergency department) and hospital admissions are documented in the study data tracking system and are reported as adverse events. Crosschecking will be done by comparing doctor reported health consumption and patient-reported health consumption.

Study participants can leave the study at any time for any reason if they wish to do so without any consequences. The local principal investigator can decide to withdraw a teenager from the study for urgent medical reasons. Another patient from the same center may then be randomized to enter the study.

Patients withdrawn from the experimental arm of the trial will continue with usual care, outside the scope of this study, as would have happened otherwise. Patients who receive usual care and withdraw from the study will continue to receive usual care as before entering the study.

### Interventions—adherence

In this trial adherence refers to the degree to which patients and IBD-team act in accordance with the flarometer advice. Low adherence can have a substantial effect on statistical power and interpretation of the trial results. To help avoid these potential detrimental effects of non-adherence, we have implemented the following procedures: (1) screening candidate participants for having an unfavourable disease course, as this may be a proxy for non-adherence; (2) reminding study participants automatically that the next flarometer score is due; (3) contacting non-responders by telephone after two weeks of passivity; (4) informing both patient and local IBD-team about the flarometer treatment advice; (5) documenting ignored red alerts (i.e. failing to intensify treatment despite flarometer score in high risk stratum); and (6) restricting the statistical analyses to those participants who replied to more than 80 % of the alerts to use the flarometer.

### Outcomes

The primary endpoint is the frequency of relapse rate during a 52-week follow-up period. Investigators, health providers and patients cannot be blinded to the assigned intervention. To minimize bias the primary endpoint is strictly defined as a clinical activity index score >10 points (see Table 1), necessitating steroid therapy, a six-week course of exclusive enteral nutrition, aminosalicylate dose escalation, or introduction of anti-TNF antibodies (infliximab or adalimumab). We will calculate Kaplan-Meier curves for both assignment groups. Time-to-relapse will be compared with a two-sided logrank test. The hazard ratio with its 95 % confidence interval for relapse will be provided with a Cox proportional hazards (multivariate) regression analysis. Potential confounding factors, including age at diagnosis, type of IBD, date of last relapse, emotional maturity and level of education, will be included in the Cox proportional hazards model.

**Table 1:** Translation of clinical score systems to Physician’s Global Assessment

Physician Global Assessment	PCDAI [7]	shPCDAI [14, 38]	PUCAI [8]
Remission	<10	<10	<10
Mild activity	10-30	10-25	10-34
Moderate activity	≥30	26-40	35-64
Severe activity		>40	≥65

**Fig. 3:**
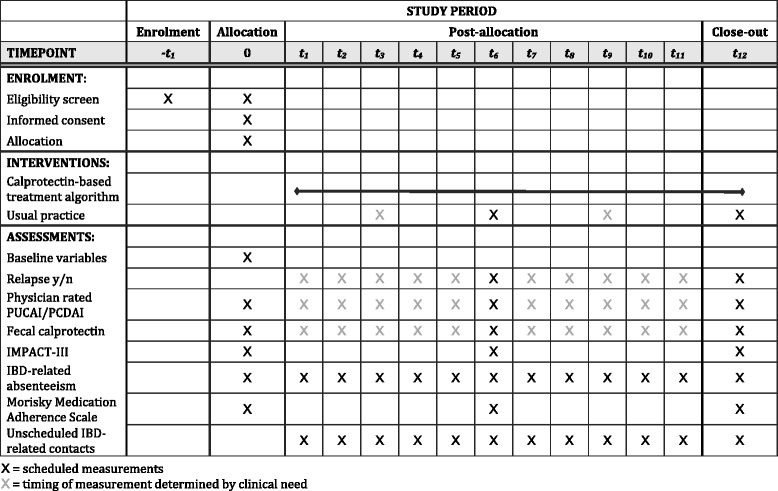
IBD-live schedule of enrollment, interventions, and assessments. Comparability between groups will be assessed by summarizing emotional maturity (Bar-On EQI:JV), quality of life (IMPACT-III), and drug adherence (Morisky Medication Adherence Scale, MMAS). All of these questionnaires will be completed at home within one week after enrollment

We are collecting data from a range of secondary endpoints, including the IMPACT-III score. This is a disease-specific quality of life score, composed of 35 items on 6 domains: IBD-related symptoms (7 items), systemic symptoms (3), emotional functioning (7), social functioning (12), body image (3) and treatment/intervention-related concerns (3) [25, 26]. Each item is scored on a 5 point Likert scale, coded from 0 to 4 points. Higher scores indicate better quality of life. The IMPACT questionnaire is validated for use in children eight years old and older and is recommended for the evaluation of new therapies because of its high sensitivity to change. The IMPACT-III (NL) is a translated and modified version of the original Canadian questionnaire that used a visual analogue scale [27, 28]. The Likert scale was introduced as it has been shown that children consider it easier to complete than a visual analogue scale [29]. To compare the difference of IMPACT-scores from baseline to final visit Student’s *t*-test or analysis of variance will be used where appropriate. Other secondary endpoints include the number of unscheduled health visits, hospital admissions, IBD-related absenteeism from school or parental work, and drug adherence, using the Morisky Medication Adherence Scale (Fig. 3) [30].

The outcome parameter of the economic evaluation is the number of relapses within one year. With this outcome parameter, the incremental cost-effectiveness ratio provides information to guide preference of the web-assisted calprotectin-based treatment algorithm over usual care. The time horizon of the evaluation will be from randomization to the end of the study. Uncertainty surrounding cost-effectiveness ratio will be explored by bootstrap replication and by varying the major cost components. Discounting will *not* be applied. The economic evaluation will be performed from a societal perspective, incorporating costs of travel to the hospital and costs of leave from work of the parents, as well as direct medical costs of IBD care (costs of medication, outpatient visits). Questionnaires will be used to collect data regarding health care consumption, travel and time costs and productivity loss at study entry and at one-month intervals thereafter. Other relevant data regarding health care consumption such as number of contacts or visits with the local IBD team, unscheduled health visits, hospital admissions, and drug use will be recorded or collected from existing hospital databases. Unit prices will be determined according to Dutch guidelines. Standard prices will be used if available and applicable, according to the type of hospital where the patient is treated. Productivity loss will be calculated using the friction cost method. The IMPACT-III score will be used in the patient outcome analysis and provides an estimate of the relationship between the difference in quality of life and the difference in costs of both treatments. At the end of the observation period we will evaluate the teenagers and caretakers in the intervention group for their attitude towards E-health.

### Other study parameters

Emotional maturity may be a risk factor for poor adherence and is therefore assessed at the start of the study using the Bar-On Emotional Quotient Inventory (Youth Version) [31]. Emotional maturity as well as the level of education of patients and parents are to be used as covariables in the final data analyses. Stool samples will also be tested for colon pathogens (*Salmonella enterica, Campylobacter jejuni*, Shigella spp/EIEC, *Giardia lamblia*, STEC) and parasites (*Cryptosporidium* spp, *Dientamoeba fragilis*, *Entamoebe histolytica*) with the real-time multiplex PCRs, to control for false positive fCal values [32, 33].

### Participant timeline

Teenagers who are potentially eligible based on inclusion criteria will be identified and informed about the trial by the local IBD team. In case the teenager is interested in participation an information pack will be sent by mail, containing a patient invitation letter, a participant information sheet and consent forms. Patients who do not participate due to exclusion criteria or refusal will be anonymously recorded, including patient characteristics and, if available, the reason for non-participation.

During the outpatient appointment the health provider will (1) recheck whether the participant has daily access to a smartphone or tablet or an internet connection, (2) assess whether the last relapse was more than three months before, and (3) identify parents or legal guardians. After written informed consent has been obtained, but before randomization occurs, each patient will undergo a detailed assessment (Fig. 3). Data collected will include demographic information (including travel time to clinic, level of education of both patient and parents), clinical and IBD-specific history (including age at diagnosis, type of IBD, disease location, date of last relapse and current maintenance treatment). The (pediatric) gastroenterologist will perform a general physical examination (including measurements of height and weight, and assessment of pubertal maturation) and will rate the current disease activity by using the PCDAI or PUCAI.

Irrespective of group assignment, each participant will be evaluated at the local trial site at six and twelve months for disease activity (by physician rated PUCAI or PCDAI), serological markers of inflammation and fCal. Each participant will complete a diary for school-absenteeism and parental absence from work once every month, and IMPACT-III and MMAS questionnaires at six and twelve months. Reminders will be sent by email, with a direct link to the online questionnaires.

In between scheduled health visits teenagers assigned to the experimental group receive alerts to use the online flarometer and to send a feces sample by pre stamped return envelope for fCal rapid analysis. Teenagers assigned to usual care may have outpatient appointments at three and nine months, depending on the health check interval prior to participation.

### Sample size

In earlier versions of the study protocol (v1 to v5) the primary outcome was defined as the number of relapses per group during a 52-week follow-up. The nul-hypothesis was based on a relapse rate of 40 % in the control group [34], whereas the alternative hypothesis stated that in the experimental group this percentage would drop down to 25 % [13]. We wished to detect this difference by a two-sided test at 5 % level of significance with a probability of 80 %. With the binary outcome (event or not) 152 patients per group were needed.

Accumulating data from the study as it continued allowed re-estimating the sample size. Adapting the primary outcome from ‘number of events’ to ‘time-to-event’ per group could reduce the required sample size. To detect a 15 % reduction in the absolute relapse risk after 52 weeks of follow-up with a two-sided significance level of 5 % and with 80 % power, we calculated that we need 90 patients per group (taking into account a maximum of 10 % loss-to-follow-up).

### Randomization and masking

Eligible patients for whom consent or assent is provided will be allocated in a 1:1 ratio to the two arms of the study according to a computer-generated random sequence stratified by research site and disease type (Crohn’s disease vs. ulcerative colitis), and using blocks of variable size. The allocation sequence is generated by the biostatistics unit of the UMCG, and is not available to any member of the research team. Allocation concealment will be ensured, as the study website (https://www.ibd-live.nl) will not release the randomization code until the teenager has been recruited into the trial, which takes place after all baseline measurements have been completed. The nature of the intervention does not allow blinding of participants, care providers, or outcome assessors.

### Retention

Once a teenager is randomized, the study site will make every reasonable effort to follow the patient for the entire study period of 52 weeks. It is projected that the rate of loss-to-follow-up will be at most 10 %. Retention will be promoted by sending automated reminders to the participants in the experimental study arm to use the flarometer, careful planning of outpatient appointments to avoid school calendar breaks, and facilitating stool collection with feces collection papers. Questionnaires will be completed digitally via a hyperlink sent by email, which will further limit participant burden.

There will be no explicit incentive for study participants. Participation in a child friendly website may be perceived as an incentive, but it is not an incentive in a material sense. Teenagers in the experimental arm will have access to the website, while the controls will only have access to the study questionnaires.

A participant who withdraws consent for follow-up assessment of any of the secondary outcomes will be given the option to continue with assessments for the primary outcome.

### Confidentiality and data management

Consecutive patients participating in the study will receive a unique study number. All data will be entered electronically and stored linked to this study number. Patients will complete digital questionnaires via a hyperlink sent by email, which will automatically be linked to the corresponding study number. Patients with access to the IBD-live website will use their username and password for identification/login. The username will be linked to the unique study number in order to store all data concerning flarometer results.

Local trial sites will have access to the flarometer data of their own patients only. The coordinating investigator in the UMCG and the webmaster are the sole persons who have access to the full subject identification code list in order to be able to link patient login-ID to the corresponding study number.

Feces samples will be marked with a study number label and sent to the Department of Laboratory Medicine at the UMCG. fCal results will be uploaded on the website by the coordinating investigator and will be visible to the individual patients and their local IBD team. After measurement of fCal levels the residual material will be analyzed for detection of microbial gut pathogens.

Data will be stored during the study period and until 15 years thereafter. When patients and their parents give permission, residual feces will be stored for a maximum period of 15 years for future diagnostic research.

### Statistical methods

The statistical analysis will be coordinated by a statistician from the biostatistics unit of the UMCG. The primary analyses will be conducted using an intention-to-treat basis. All teenagers recruited into the study will be included in these analyses; patients will be analyzed within the group (IBD-live or usual practice) to which they were randomized irrespective of what care they actually received. Where appropriate, secondary analyses will be conducted using a per protocol basis. These analyses will be restricted to just those teenagers who replied to more than 80 % of the alerts to use the flarometer and to the teenagers in the control group who replied to more than 80 % of the requests to send a stool sample for fCal measurement.

As earlier published web-based programs have focussed on patients with ulcerative colitis [17, 35, 36], we made an a priori decision to do a subgroup analysis to examine the intervention in two subtypes of IBD (ulcerative colitis and Crohn’s disease). We will use a test of interaction to examine whether the treatment effect differs between these subgroups. In case of random missing data multiple imputation will be performed using the hot deck method [37].

### Data monitoring

A Data Safety Monitoring Board (DSMB) has been established. The Chair of the DSMB is Dr. Nic Veeger (epidemiologist at the University Medical Centre Groningen), with Dr. Hans Burgerhof (statistician, UMCG), and Dr. Gieneke Gonera-de Jong, paediatrician at Wilhelmina Hospital Assen). The Charter and responsibilities of the DSMB are available on request from the IBD-live study office. The DSMB is independent of the study organizers. During the period of recruitment to the study, one interim analysis will be supplied, in strict confidence, to the DSMB, together with any other analyses that the committee may request. In the light of this interim analysis, the DSMB will advise the trial steering committee if, in its view, the active intervention has been proved, beyond reasonable doubt, to be different from the control (usual practice) for all participants. The trial steering committee can then decide whether or not to modify intake to the trial. The advice of the DSMB will only be sent to the sponsor of the study. Should the sponsor decide not to fully implement the advice of the DSMB, the sponsor will then send the advice to the reviewing Medical Ethical Committee (MEC), including a note to substantiate why (part of) the advice of the DSMB will not be followed.

### Premature termination of the study

An interim safety analysis will be performed after the first 50 participants in both arms have completed the first six months of the study. The interim-analysis will be performed by the trial statistician. This will be based on the number of clinical relapses, a significant difference in IBD-associated hospital admissions and/or emergency visits and/or unscheduled health visits, to the detriment of the intervention group (IBD-live). As we expect a non-normal distribution, the Mann–Whitney *U* test will be used to test the differences that may have occurred. As it may be hard to show a statistically significant difference due to the sample size we propose a composite-core as proxy for severity of clinical deterioration of IBD during the study, based on objective parameters. In this score an unscheduled health visit = 1 point, an emergency visit = 2 points, and a hospital admission = 4 points. Given a sample size of n = 50, an α of 0.05 (two-tailed) and an estimated standard deviation of 4 points, we can reliably show a difference of 3 points on the composite-score, which equals one hospital admission.

### Harms

This trial should be regarded as therapeutic research, as two different treatment strategies are compared. Patients allocated to usual care will receive IBD care as before. There is no additional risk in this group. Patients in the experimental arm will have fewer scheduled encounters with their local IBD team, and blood drawings will be reduced to once every six months. This relatively long interval between two phlebotomies may hinder early recognition of adverse drug reactions, although it is not uncommon for teenagers with quiescent IBD to have only two blood drawings per year. Patients from both groups are instructed to contact the local IBD-team if they experience relapse, or if at any time they want a consultation. The management of relapses is not changed from the current routine practice and is the same for both groups.

In this trial an adverse event will be defined as any undesirable experience occurring to a participant during the study, whether or not considered related to the experimental intervention, including any unscheduled health visit. Adverse events will be collected after the participant has provided consent and enrolled in the study. If an adverse event is experienced within one month after the enrollment visit, the event will be reported as not related to the trial. All adverse events occurring later than one month after enrollment will be recorded. An adverse event that meets the criteria for a serious adverse event between study enrollment and hospital discharge will be reported to the local MEC.

### Ethics

The trial will be conducted according to the principles of the Declaration of Helsinki (59th version, October 2008) and in accordance with the Dutch Medical Research Involving Human Subjects Act (WMO). The independent Medical Ethical Committee (MEC) of the University Medical Centre Groningen (Groningen, the Netherlands) has approved the study protocol.

Secondary approval was obtained from the boards of the Academic Medical Centre (Amsterdam), Erasmus Medical Centre (Rotterdam), Haga Hospital (Den Haag), Isala Hospital (Zwolle), Leiden University Medical Centre (Leiden), Medisch Spectrum Twente (Enschede), Rijnstate Hospital (Arnhem), University Medical Centre Utrecht (Utrecht), and VU medical center (Amsterdam) according to the Dutch CCMO External Review Directive 2012 (RET 2012).

Any modifications to the protocol which may impact on the conduct of the study, potential benefit of the patient or may affect patient safety, including changes of study objectives, study design, patient population, sample sizes, study procedures, or significant administrative aspects will require a formal amendment to the protocol. Such amendment will be approved by the MEC prior to implementation and notified to the participating centers.

Written informed consent will be obtained from both parents or legal guardians of all participants younger than 18 years old, and from all teenagers 12- to 19-years old, prior to randomization. The trial is registered in the Dutch Trial Register (http://www.trialregister.nl/trialreg/index.asp) with identification number NTR3759.

## Discussion

This trial will test the use of remission induction therapy delivered according to a web-assisted calprotectin-based treatment algorithm head-to-head against the same treatment based on physician assessment of individual patient characteristics.

We expect that use of IBD-live will result in better disease control, expressed as a lower relapse rate, accompanied by lower costs of disease management as compared to usual care. The number of scheduled outpatient visits is expected to drop by 50 %, unscheduled visits for uncertainty concerning suspected relapse will probably reduce in number, and the number of unscheduled visits for confirmed relapse are also expected to drop by 50 %. The physician’s workload is expected to decrease, while the nurse’s workload is expected to increase. Combined with the expected drop in relapse frequency, this could mean that IBD-live is the dominant strategy in the cost-effectiveness analysis.

### Relevance for practice/implementability

IBD-live care corresponds to the needs and wishes of both patients and clinicians. Unnecessary, but time-consuming visits to the clinic are reduced, while the quality of disease monitoring is maintained or even improved. Scientific societies and patient associations broadly support the concept of tailor-made IBD-management, and health insurance companies are co-financing this scientific project.

If IBD-live care proves to be effective, funding for this type of long-term care will be provided under the Exceptional Medical Expenses Act (AWBZ). The trial will yield information on disease monitoring with fCal, which has hitherto been a gap in both national and international guidelines. Study results will eventually be included in Dutch and international guidelines.

### Additional value of the study

Pediatricians have so far always been reluctant to allow teenagers in the driving seat. Now that reliable non-invasive predictors of relapse have become available, home monitoring may be a feasible concept. If IBD-live care proves to be effective, this would mean a fundamental change in approach.

Treatment intensification in teenagers who are doing well, but who have a high risk of progression to clinically overt relapse, is currently not recommended. Periodically measuring fCal levels may facilitate recognition of preclinical relapse. The trial described in this protocol will allow us to determine whether preemptive treatment of teenagers with increased fCal levels, but who report no complaints, really prevents progression to symptomatic relapse. This could be the second paradigm shift.

### Trial status

Recruitment began on 6 June 2013. It is anticipated that study recruitment will be completed by 30 September 2015, and that the trial will conclude by 30 September 2016.

## Abbreviations

CRP: C-reactive protein
DSMB: Data Safety Monitoring Board
fCal: Fecal calprotectin
IBD: Inflammatory bowel disease
MEC: Medical Ethical Committee
MMAS: Morisky Medication Adherence Scale
PCDAI: Pediatric Crohn’s Disease Activity Index
PUCAI: Pediatric Ulcerative Colitis Activity Index
shPCDAI: Short version of the PCDAI
TNF: Tumor necrosis factor
UMCG: University Medical Centre Groningen
